# Exposure to Neighborhood Racialized Economic Segregation and Reinjury and Violence Perpetration Among Survivors of Violent Injuries

**DOI:** 10.1001/jamanetworkopen.2023.8404

**Published:** 2023-04-26

**Authors:** Elizabeth C. Pino, Sara F. Jacoby, Elizabeth Dugan, Jonathan Jay

**Affiliations:** 1 Department of Emergency Medicine, Boston Medical Center, Boston University School of Medicine, Boston, Massachusetts; 2 Penn Injury Science Center, University of Pennsylvania, Philadelphia; 3 Boston Violence Intervention Advocacy Program, Department of Emergency Medicine, Boston Medical Center, Boston, Massachusetts; 4 Department of Community Health Sciences, Boston University School of Public Health, Boston, Massachusetts

## Abstract

**Importance:**

Much is unknown about how individual and neighborhood factors converge in the association with risk for violent reinjury and violence perpetration.

**Objectives:**

To investigate the association of exposure to neighborhood racialized economic segregation with reinjury and use of violence against others among survivors of violent penetrating injury.

**Design, Setting, and Participants:**

This retrospective cohort study was performed using data obtained from hospital, police, and state vital records. The study was performed at Boston Medical Center, an urban, level I trauma center that is the largest safety-net hospital and busiest trauma center in New England. The cohort included all patients treated for a nonfatal violent penetrating injury from 2013 to 2018. Patients with no Boston metropolitan area home address were excluded. Individuals were followed up through 2021. Data were analyzed from February to August 2022.

**Exposure:**

American Community Survey data were used to measure neighborhood deprivation using the racialized economic Index of Concentration at the Extremes (ICE) for patient residential address upon hospital discharge. ICE was measured on a scale from −1 (most deprived) to 1 (most privileged).

**Main Outcomes and Measures:**

Primary outcomes were violent reinjury and police-reported perpetration of violence within 3 years of an index injury.

**Results:**

Of 1843 survivors of violence (median [IQR] age, 27 [22-37] years; 1557 men [84.5%]; 351 Hispanic [19.5%], 1271 non-Hispanic Black [70.5%], and 149 non-Hispanic White [8.3%] among 1804 patients with race and ethnicity data), the cohort was skewed toward residing in neighborhoods with higher racialized economic segregation (median [IQR] ICE = −0.15 [−0.22 to 0.07]) compared with the state overall (ICE = 0.27). There were police encounters for violence perpetration among 161 individuals (8.7%) and violent reinjuries among 214 individuals (11.6%) within 3 years after surviving a violent penetrating injury. For each 0.1-unit increase in neighborhood deprivation, there was a 13% (hazard ratio [HR], 1.13; 95% CI, 1.03 to 1.25; *P* = .01) increase in risk of violence perpetration but no difference in risk for violent reinjury (HR, 1.03; 95% CI, 0.96 to 1.11; *P* = .38). The greatest occurrence for each outcome was within the first year after index injury; for example, incidents of violence perpetration occurred among 48 of 614 patients (7.8%) at year 1 vs 10 of 542 patients (1.8%) at year 3 in tertile 3 of neighborhood deprivation.

**Conclusions and Relevance:**

This study found that living in a more economically deprived and socially marginalized area was associated with increased risk of using violence against others. The finding suggests that interventions may need to include investments in neighborhoods with the highest levels of violence to help reduce downstream transmission of violence.

## Key Points

**Question:**

Is the level of residential racialized economic segregation associated with the risk of violent reinjury and police encounters for violence perpetration among survivors of violent injuries?

**Findings:**

In this cohort study that included 1843 survivors of violent penetrating injuries, there was an association between the level of racialized economic segregation in the neighborhood to which a patient returned after hospital discharge and the risk for future use of violence against others. The level of neighborhood deprivation was not associated with reinjury risk.

**Meaning:**

This study found that racialized poverty in urban communities was associated with adverse outcomes among survivors of violent injuries, suggesting the importance of addressing the root causes of violence.

## Teaser

This cohort study investigates the association of racialized economic segregation with the risk of reinjury and violence perpetration among survivors of violent injuries.

## Introduction

Violence-related injuries are a critical public health issue in US cities. Among city-dwelling individuals in the US ages 15 to 44 years, homicide is the second leading cause of death.^1^ For every violence-related injury that results in death, several more injuries are nonfatal.^2^ Hospital visits for the most serious violence-related injuries have increased in number since the start of the COVID-19 pandemic.^3^ Before and during the pandemic, there were clear and extreme disparities in the spatial distribution of community violence by race and ethnicity, with Black and Hispanic or Latinx communities experiencing disproportionate exposure.^4^

Individuals who survive violence-related injuries often experience mental and physical health consequences, including posttraumatic stress, anxiety, depression, functional limitations, and sometimes serious disability.^5,6,7^ After an individual survives a violence-related injury, there is a heightened risk of experiencing violence again or using violence against others.^8,9^ There is no single explanation underlying the risks for reinjury. The lives to which individuals who experienced violence return after hospitalization are often complicated and unpredictable^10^; a multitude of factors, which may be individual, relational and environmental in etiology, may be associated with reinjuries.^11^ In the context of a commonly cited contagion theory of violence, an initial injury may represent the effects of ongoing conflict between individuals or groups and may be associated with increased conflict or new conflicts.^12^

Tertiary prevention programs attend to the postinjury phase as an important opportunity to offer services to address the mental and physical trauma experienced by survivors.^13,14^ A prevailing rationale for these hospital-based violence-intervention programs (HVIPs) is that offering social services in the postinjury period may leverage a window of opportunity when survivors may be motivated to change behaviors (eg, substance use), given the opportunity to develop new skills, provided opportunities for economic stability (eg, steady employment), and readily engaged in therapeutic modalities to address the experience of traumatic stress.^15^ Promising evidence supports the effectiveness of these programs.^16,17,18,19,20,21,22^ However, a substantial proportion of eligible patients decline services (eg, 63% in the setting where our study took place),^23^ suggesting that HVIPs may be part of the solution but not the sole intervention needed to prevent future violence experienced by individuals who were previously injured. Whether or not survivors accept services, communities to which they return after hospitalization may also be associated with increased risks for injuries, especially when located in geographies where economic disinvestment, poverty, intergenerational trauma, and structural racism reinforce the persistence of community violence hot spots.^15,24^

In addition to HVIPs, multilevel strategies that focus on community and neighborhood-level factors associated with violent reinjury are critically needed.^24^ Most prior work has focused on individual-level factors, such as substance use and attitudes and behaviors associated with violence-related norms.^25,26^ These are important but wholly insufficient to address the risks for being reinjured by violence contingent to an individual’s residential environment.^27^ In US cities, residential racial segregation is a fundamental cause of health disparities because it enables the place-based stratification of social goods needed for health (eg, schools, parks, and jobs).^28^ In particular, the spatial differentiation of neighborhoods by race and ethnicity and income (hereafter *racialized economic segregation*) has been found to be a powerful factor for estimating community-level firearm violence rates.^29^ No previous study, to our knowledge, has examined the association of racialized economic segregation with postinjury, violence-related outcomes.

The objective of this study was to estimate the association of neighborhood-level factors, specifically racialized economic segregation, with reinjury and police encounters for violence perpetration within 3 years of an initial violence-related injury. This retrospective study was performed using a cohort of patients presenting to the Boston Medical Center (BMC) for a violent penetrating injury from 2013 to 2018. We hypothesized that patients who were discharged to residential environments in areas with the highest relative social and economic deprivation would experience greater rates of reinjury and be more likely to carry out violence within the follow-up period.

## Methods

The Boston University BMC institutional review board approved this cohort study with a waiver of Health Insurance Portability and Accountability Act of 1996 authorization for research and deemed this study exempt from federal regulations for the protection of human research participants. Informed consent was waived by Boston University because it was deemed impracticable for retrospectively collected clinical data, given the large sample size and difficulty of locating patients. Results were reported according to the Strengthening the Reporting of Observational Studies in Epidemiology (STROBE) reporting guideline

### Study Design and Population

This retrospective study was performed using data from a cohort of patients presenting to the BMC emergency department for a violent penetrating injury between 2013 and 2018. BMC, the region’s largest safety-net hospital, is a level I trauma center that treats approximately 70% of patients with gunshot and stab wounds in the city of Boston.^30^ Patients were identified from the BMC Violence Intervention Advocacy Program clinical database, as previously described.^23,31^ Patients included in this analysis were monitored for outcomes through 2021, allowing at least 3 years of follow-up. Additional details of the study design are presented in eAppendix 1 and eFigure 1 in Supplement 1.^13^

### Measures

Variables for patient demographics and injury characteristics are described in eAppendix 2 in Supplement 1. We abstracted self-identified race and ethnicity from the patient medical record. Race and ethnicity were classified into 5 categories: non-Hispanic White, non-Hispanic Black, Hispanic (any race), any other race (including Asian, American Indian or Alaskan Native, Native Hawaiian or Pacific Islander, and all other races), and those missing race and ethnicity information (“unknown”). Race and ethnicity were queried in 2 separate questions and combined by including Hispanic individuals of any race in the Hispanic category. The primary exposure of interest was the relative social and economic context of patient residential environment as measured by the Index of Concentration at the Extremes (ICE) for race and ethnicity and for income by census tract. This measure captures racialized economic segregation, or the polarization of neighborhoods by extremes of social privilege (high income and White race) and deprivation (low income and Black race). The index has a range of −1 (most deprived) to 1 (most privileged).^32,33^ The ICE scale has been used in multiple studies of the distribution of health outcomes.^29,33,34,35^ Racialized economic segregation is the intentional product of historical and ongoing actions, including redlining, block busting, and gentrification, by governmental and private entities; each of these actions has reinforced the spatial separation of racial and ethnic groups and differential access to economic resources, such as well-paying jobs and high-quality education.^28,36,37,38,39,40^ Conceptually, this measure is better able to detect the variation in privilege that occurs at low levels of deprivation compared with similar measures.^41^ Prior work found that the race and ethnicity–income ICE estimated community violence better than race and ethnicity or income alone^29^; the race and ethnicity–income ICE may be the single best factor estimating neighborhood-level violence rates.^42^ Calculation of patient deprivation exposure is described in eAppendix 3 in Supplement 1.^43^

The primary outcomes of this study were violent reinjury and police-reported violence perpetration (eAppendix 4 in Supplement 1). Time to event was calculated for each event from the date of the initial nonfatal penetrating injury treated at BMC to the date of alleged perpetration of violence or violent reinjury. The follow-up time for each patient was determined by the date of the initial nonfatal penetrating injury to the death date or the last day of the year 2021 for patients surviving the study period.

### Statistical Analysis

We compared baseline patient and injury characteristics by neighborhood deprivation tertile. Categorical variables were compared using χ^2^ tests or the Fisher exact test. Continuous variables were compared using the nonparametric Kruskal-Wallis equality-of-populations rank test after rejection of the assumption of normality using the Shapiro-Francia test.^44^ Variables with missing information (ie, unknown values) were included in the analysis, and data imputation was not used to replace missing values.

We then used Cox proportional hazard regression models to estimate hazard ratios (HRs) and 95% CIs. In regression models, ICE values were assessed as a continuous variable. HRs are reported as the change in risk per 0.1-unit change in ICE racialized economic segregation on a scale from 1 to −1 (ie, the equivalent of an increase in the proportion of neighborhood households with race and ethnicity–income deprivation by 10 percentage points or a reduction in privileged households of the same magnitude). A detailed explanation of the calculation of the ICE scale for income and race and ethnicity is described in Feldman et al.^33^ Regression model development and assessment of residual autocorrelation are described in eAppendix 5 in Supplement 1.^42,45,46^ Data were analyzed from February to August 2022. Analyses were conducted using R statistical software version 4.2.2 (R Project for Statistical Computing) and Stata statistical software version 16 (StataCorp), and maps were constructed in ArcGIS Pro version 3.1 (Esri). Statistical tests used 2-sided *P* < .05 as the threshold for significance.

## Results

**Figure 1.**
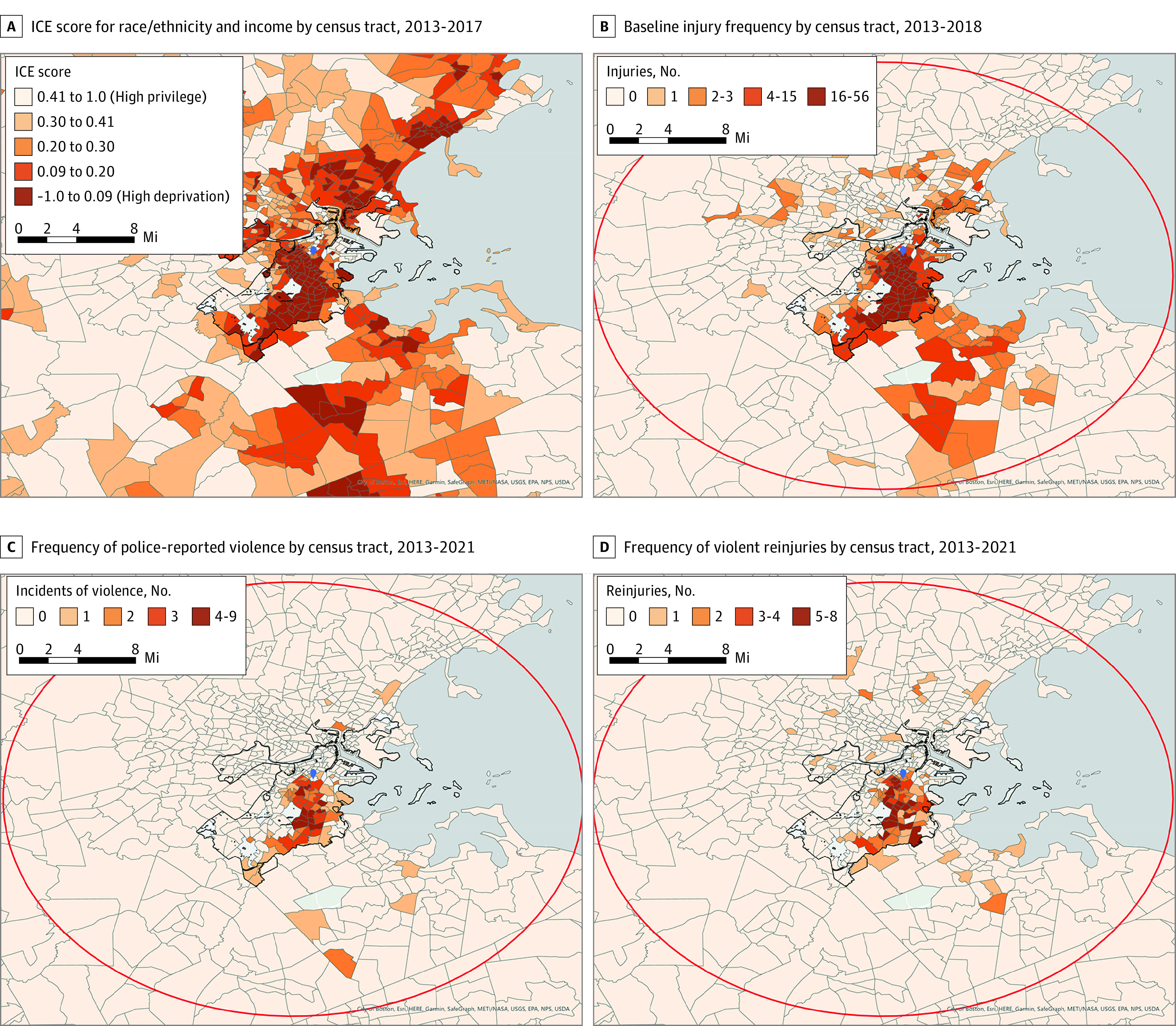
Racialized Economic Segregation and Residences of Survivors of Violence by Census Tract. Marker indicates the location of Boston Medical Center; ICE, Index of Concentration at the Extremes; red circle, 15-mile radius from the geographic center of Boston; thicker black line, City of Boston boundary.

There were 1843 patients (median [IQR] age, 27 [22-37] years; 1557 men [84.5%] and 286 women [15.5%]; 351 Hispanic [19.5%], 1271 non-Hispanic Black [70.5%], and 149 non-Hispanic White [8.3%] among 1804 patients with race and ethnicity data) included for analysis who were treated for a violent penetrating injury at BMC from 2013 to 2018. Overall, there were 615 patients (33.3%) in ICE tertile 1 (T1), 614 patients (33.3%) in ICE tertile 2 (T2), and 614 patients in ICE tertile 3 (T3). After inverse distance weighting, address-level deprivation scores ranged from −0.4 to 0.6 (median [IQR], −0.15 [−0.22 to 0.07]). Figure 1A displays census tract–level deprivation scores for the city of Boston and surrounding areas, revealing clusters of privilege and deprivation; in the state overall, census tract deprivation scores ranged from −0.4 to 1.0 (overall ICE for Massachusetts = 0.27). The baseline patient population of individuals who experienced violence was skewed toward residing in neighborhoods with higher ICE deprivation scores than the median value for the state (Figure 1B; eFigure 2 in Supplement 1).

Patient demographics, baseline comorbidities, and neighborhood and injury characteristics differed by level of neighborhood deprivation (Table 1). Compared with patients living in areas of higher privilege, those residing in areas with higher levels of deprivation experienced violent injury at a younger median (IQR) age (T1: 29 [23-38] years; T2: 26 [22-36] years; T3: 27 [22-36] years; *P* = .01) and were more likely to identify as Black (T1: 318 of 598 patients with data [53.2%]; T2: 470 of 602 patients with data [78.1%]; T3: 483 of 604 patients with data [80.0%]; *P* < .001) than Hispanic or White. Preinjury cocaine (T1: 70 patients [11.4%]; T2: 40 patients [6.5%]; T3: 48 patients [7.8%]; *P* = .007) or opioid (T1: 49 patients [8.0%]; T2: 11 patients [1.8%]; T3: 23 patients [3.8%]; *P* < .001) use disorders were more prevalent among patients from areas of lower deprivation. As the level of deprivation increased, patients were more likely to be injured by firearm violence (T1: 242 patients [39.4%]; T2: 261 patients [42.5%]; T3: 291 patients [47.4%]; *P* = .02) than stabbing violence.

**Table 1.** Baseline Patient and Injury Characteristics, 2013-2018

Characteristic	Index of Concentration at the Extremes score				*P* value, overall (excluding unknown)^a^
	Overall (N = 1843)	T1: higher privilege (n = 615 [33.3%])	T2: 614 (n = 33.3%])	T3: higher deprivation 614 (n = 33.3%])	
Deprivation index score, median (IQR)	−0.15 (0.22 to 0.07)	0.16 (0.07 to 0.26)	−0.15 (−0.18 to −0.11)	−0.25 (−0.27 to −0.22)	>.001
Age, y					
Median (IQR)	27 (22 to 37)	29 (23 to 38)	26 (22 to 36)	27 (22 to 36)	.01
Group, y					
≤26	943 (51.2)	284 (46.2)	330 (53.8)	329 (53.6)	.01
≥27	900 (48.8)	331 (53.8)	284 (46.3)	285 (46.4)	
Gender					
Women	286 (15.5)	82 (13.3)	108 (17.6)	96 (15.6)	.12
Men	1557 (84.5)	533 (86.7)	506 (82.4)	518 (84.4)	
Race and ethnicity					
Total with data, No.	1804	598	602	604	NA
Black	1271 (70.5)	318 (53.2)	470 (78.1)	483 (80.0)	<.001 (<.001)
Hispanic	351 (19.5)	147 (24.6)	105 (17.4)	99 (16.4)	
White	149 (8.3)	114 (19.1)	17 (2.8)	18 (3.0)	
Other^b^	33 (1.8)	19 (3.2)	10 (1.7)	4 (0.7)	
Unknown, No.	39	17	12	10	
Insurance payer					
Total with data, No.	1430	470	481	479	NA
Medicaid, Medicare, or other	1075 (75.2)	343 (73.0)	362 (75.3)	370 (77.2)	.79 (.66)
Private	159 (11.1)	58 (12.3)	52 (10.8)	49 (10.2)	
No health insurance	196 (13.7)	69 (14.7)	67 (13.9)	60 (12.5)	
Unknown, No.	413	145	133	135	
Employment status					
Total with data, No.	1353	432	461	460	NA
Employed	639 (47.2)	219 (50.7)	219 (47.5)	201 (43.7)	.06 (.11)
Unemployed	714 (52.8)	213 (49.3)	242 (52.5)	259 (56.3)	
Unknown, No.	490	183	153	154	
Comorbidity					
Any mental health diagnosis	216 (11.7)	69 (11.2)	70 (11.4)	77 (12.5)	.74
Any substance use or abuse disorder	365 (19.8)	134 (21.8)	113 (18.4)	118 (19.2)	.30
Alcohol	272 (14.8)	102 (16.6)	82 (13.4)	88 (14.3)	.26
Cocaine	158 (8.6)	70 (11.4)	40 (6.5)	48 (7.8)	.007
Heroin or other opioid	83 (4.5)	49 (8.0)	11 (1.8)	23 (3.8)	<.001
Injury specifics					
Type					
Stab wound	1049 (56.9)	373 (60.7)	353 (57.5)	323 (52.6)	.02
Gunshot wound	794 (43.1)	242 (39.4)	261 (42.5)	291 (47.4)	
Hospital disposition					
Total with data, No.	1831	610	611	610	NA
Admitted	1025 (56.0)	336 (55.1)	347 (56.8)	342 (56.1)	.83
Discharged	731 (39.9)	250 (41.0)	242 (39.6)	239 (39.2)	
Eloped	75 (4.1)	24 (3.9)	22 (3.6)	29 (4.8)	
Missing, No.	12	5	3	4	
Admitted patient characteristics, median (IQR)					
ISS	5 (1 to 10)	5 (1 to 10)	5 (1 to 10)	5 (1 to 10)	.80
Length of stay, d	1.8 (0.8 to 5.5)	2.1 (0.8 to 6.0)	1.7 (0.7 to 5.6)	1.8 (0.8 to 4.9)	.64
Discharge placement					
Total with data, No.	1021	334	347	340	NA
Home	862 (84.4)	272 (81.4)	304 (87.6)	286 (84.1)	.24
Rehabilitation, long-term care, or further hospitalization	104 (10.2)	36 (10.8)	31 (8.9)	37 (10.9)	
Left against medical advice	48 (4.7)	23 (6.9)	10 (2.9)	15 (4.4)	
Police custody	7 (0.7)	3 (0.9)	2 (0.6)	2 (0.6)	
Missing, No.	4	2	0	2	
Disability					
Total with data, No.	800	254	285	261	NA
None	6 (0.8)	2 (0.8)	1 (0.4)	3 (1.2)	.04 (.10)
Temporary	746 (93.5)	240 (95.2)	270 (94.7)	236 (90.4)	
Moderate	34 (4.3)	7 (2.8)	8 (2.8)	19 (7.3)	
Severe	12 (1.5)	3 (1.2)	6 (2.1)	3 (1.2)	
Unknown, No.	227	84	62	81	

Abbreviations: ISS, injury severity score; NA, not applicable; T, tertile.

^a^ *P* value excluding unknown category is shown in parentheses. Categorical variables were compared using χ^2^ tests except for discharge placement and disability, which were compared using Fisher exact test. Continuous variables were compared using the Kruskal-Wallis equality of populations rank test.

^b^ Other race and ethnicity includes Asian, American Indian or Alaskan Native, Native Hawaiian or Pacific Islander, and all other races.

There were 96 individuals with police encounters for violence perpetration (5.2%) and 112 individuals with violent reinjuries (6.1%) within 1 year after the index injury, and there were 161 individuals with incidents of alleged violence perpetration (8.7%) and 214 individuals with violent reinjuries (11.6%) within 3 years of the index injury (eTable 1 in Supplement 1; Figure 1C-D). These risks by level of neighborhood deprivation are presented in Table 2 and eTable 2 and eTable 3 in Supplement 1. In our models, for each 0.1-unit increase in neighborhood deprivation, there was a 17% increase in risk of police-reported violence perpetration at 1 year (HR, 1.17; 95% CI, 1.03-1.33; *P* = .02) and a 13% increase in such risk at 3 years (HR, 1.13; 95% CI, 1.03-1.25; *P* = .01) but no change in risk for violent reinjury at 1 year (HR, 1.03; 95% CI, 0.93-1.14; *P* = .53) or 3 years (HR, 1.03; 95% CI, 0.96-1.11; *P* = .38). Patients at highest risk for police-reported violence perpetration at 3 years were overwhelmingly men (HR, 5.95; 95% CI, 2.44-14.49; *P* < .001) and Black (vs Hispanic: HR, 2.80; 95% CI, 1.64-4.78; *P* < .001; vs White: HR, 10.60; 95% CI, 1.47-76.59, *P* < .001). Age (HR per 1-year increase, 0.93; 95% CI, 0.91-0.95; *P* < .001) and injury year (HR, 0.76; 95% CI, 0.68-0.84; *P* < .001) were inversely associated with the risk for police-reported violence perpetration. A diagnosis of substance use was associated with violent reinjury (HR, 2.38; 95% CI, 1.74-3.26; *P* < .001).

**Table 2.** Risk of Violence at 1 and 3 Years

Factor	Police-reported violence perpetration^a^			Violent reinjury^b^		
	Survivors, No. (%) (n = 1843)	Multivariable model, HR (95% CI)^c^	*P* value	Survivors, No. (%) (n = 1843)	Multivariable model, HR (95% CI)^c^	*P* value
At 1 y	96 (5.2)	NA	NA	112 (6.1)	NA	NA
ICE deprivation index score^d^	NA	1.17 (1.03-1.33)	.02	NA	1.03 (0.93-1.14)	.53
Injury year, per 1-year increase	NA	0.82 (0.72-0.94)	.003	NA	0.95 (0.85-1.07)	.41
Age, per 1-year increase	NA	0.93 (0.90-0.96)	<.001	NA	1.00 (0.98-1.01)	.70
Gender						
Women	3 (1.1)	1 [Reference]	.003	16 (5.6)	1 [Reference]	.61
Men	93 (6.0)	5.77 (1.83-18.23)		96 (6.2)	1.15 (0.68-1.95)	
Race and ethnicity						
Black	86 (6.8)	5.65 (0.77-41.31)	.04	79 (6.2)	0.78 (0.41-1.48)	.89
Hispanic	9 (2.6)	2.17 (0.27-17.22)		19 (5.4)	0.71 (0.34-1.49)	
White	1 (0.7)	1 [Reference]		13 (8.7)	1 [Reference]	
Any substance use or abuse	7 (1.9)	NA	NA	40 (11.0)	2.31 (1.50-3.54)	<.001
At 3 y	161 (8.7)	NA	NA	214 (11.6)	NA	NA
ICE deprivation index score^d^	NA	1.13 (1.03-1.25)	.01	NA	1.03 (0.96-1.11)	.38
Injury year, per 1-unit increase	NA	0.76 (0.68-0.84)	<.001	NA	0.95 (0.87-1.03)	.18
Age, per 1-unit increase	NA	0.93 (0.91-0.95)	<.001	NA	0.99 (0.98-1.00)	.16
Gender						
Women	5 (1.8)	1 [Reference]	<.001	29 (10.1)	1 [Reference]	.32
Men	156 (10.0)	5.95 (2.44-14.49)		185 (11.9)	1.22 (0.83-1.81)	
Race and ethnicity						
Black	145 (11.4)	10.60 (1.47-76.59)	<.001	164 (12.9)	0.97 (0.59-1.59)	.17
Hispanic	15 (4.3)	3.79 (0.21-28.83)		28 (8.0)	0.62 (0.34-1.11)	
White	1 (0.7)	1 [Reference]		21 (14.1)	1 [Reference]	
Any substance use or abuse	14 (3.8)	NA	NA	73 (20.0)	2.38 (1.74-3.26)	<.001

Abbreviations: HR, hazard ratio; ICE, Index of Concentration at the Extremes; NA, not applicable.

^a^ Police-reported violence perpetration included stabbing, firearm injury, blunt assault, or threats of violence that involved Boston police interaction. Threats of violence included verbal threats, threatening texts or phone messages, or attempted assault, stabbing, or shooting.

^b^ Violent reinjuries included stab wounds, gunshot wounds, or blunt assaults treated at Boston Medical Center or that involved Boston police interaction or homicides reported to the state vital statistics department.

^c^ Cox proportional hazards regression models were used to estimate HRs and 95% CIs. The multivariable model for police-reported violence perpetration was adjusted for age, gender, race and ethnicity, and year of initial injury. The multivariable model for violent reinjury was adjusted for those factors and diagnosis of a substance use disorder.

^d^ In all models, the ICE score was assessed as a continuous variable. All HRs are reported as the change in risk per 0.1-unit change in ICE racialized economic segregation on a scale from 1 to −1.

We conducted several sensitivity analyses. Regression models were reassessed after excluding 203 patients (11.0%) residing outside Boston, and results were similar in magnitude and significance (eTable 4 in Supplement 1). Variables representing residential instability and distance to a level I trauma center were added to models, and results were not changed (eTables 2, 3, and 5 in Supplement 1).

**Figure 2.**
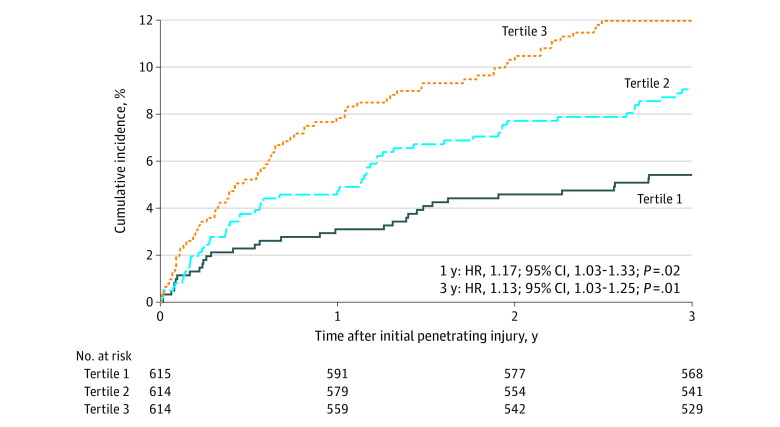
Cumulative Incidence of Police-Reported Violence Perpetration by Level of Neighborhood Deprivation, 2013-2021 The Index of Concentration at the Extremes was assessed as a continuous variable. Hazard ratios (HRs) are reported as the change in risk per 0.1-unit change in Index of Concentration at the Extremes racialized economic segregation on a scale from 1 to −1.

**Figure 3.**
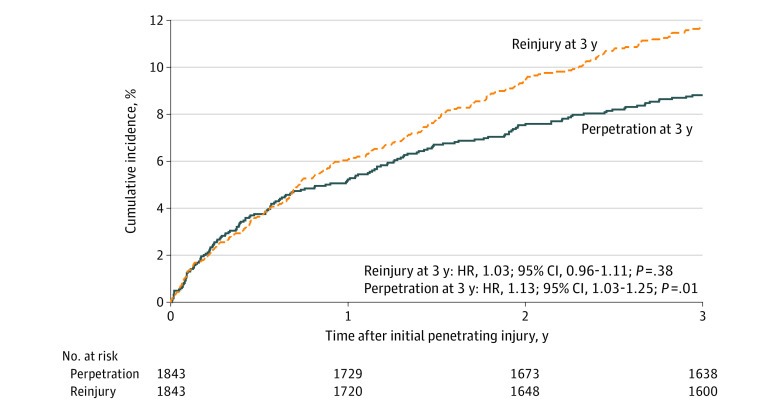
Cumulative Incidence of Police-Reported Violence Perpetration and Violent Reinjury, 2013-2021 In all models, the Index of Concentration at the Extremes was assessed as a continuous variable. Hazard ratios (HRs) are reported as the change in risk per 0.1-unit change in Index of Concentration at the Extremes racialized economic segregation on a scale from 1 to −1.

The cumulative incidence of police-reported violence perpetration and violent reinjury by neighborhood deprivation tertile are shown in Figure 2 and eFigure 3 in Supplement 1. Kaplan-Meier curves estimating the survival function for violence perpetration in comparison with violent reinjury are shown in Figure 3. These curves illustrate that the greatest occurrence of violence perpetration, particularly for patients at the highest levels of neighborhood deprivation, was within the first year after violent injury. For example, incidents of violence perpetration occurred among 48 of 614 patients (7.8%) at year 1 vs 10 of 542 patients (1.8%) at year 3 in tertile 3 of neighborhood deprivation, while they occurred among 19 of 615 patients (3.1%) at year 1 vs 5 of 577 patients (0.9%) at year 3 in tertile 1. The rate of violent reinjury remained more constant throughout the 3-year study period. For example, violent reinjuries occurred among 48 of 614 patients (7.8%) at year 1 vs 16 of 542 patients (3.0%) at year 3 in tertile 3 of neighborhood deprivation, while these events occurred among 37 of 615 patients (6.0%) at year 1 vs 11 of 577 patients (2.0%) at year 3 in tertile 1.

## Discussion

To our knowledge, this cohort study is the first study to examine the associations of individual-level factors and neighborhood-level racialized economic segregation with the risk for adverse outcomes after violent injury. This study found that for survivors of violent penetrating injury, living in neighborhoods with higher deprivation levels was associated with increased risk of police-reported violence perpetration. Contrary to our hypothesis, we found that the level of racialized economic segregation to which a survivor of violence returned after hospital discharge was not associated with reinjury risk. Most survivors of violence in our sample resided in Boston neighborhoods with higher than median levels of racialized economic segregation. Patients living in areas with the highest deprivation levels were more likely to have been injured at baseline by a firearm and at a younger age, while those residing in neighborhoods with higher privilege levels were more likely to have a preinjury diagnosis of cocaine or opioid use. Patients in our population with the highest risk for violence perpetration were younger, men, and Black. As we have previously shown,^47,48,49^ patients with a baseline diagnosis of substance use disorder were at highest risk for violent reinjury.

Our finding that racialized economic segregation was associated with violence perpetration among survivors of violent penetrating injury is consistent with results from other analyses addressing spatial health inequities and social determinants of health.^29^ The ICE for racialized economic segregation has been shown to be associated with short-term, long-term, and intergenerational health outcomes,^33,50^ as well as the likelihood of community-level assaultive injuries.^29^ However, to our knowledge our study is the first to show a direct association between racialized economic segregation and the place-based risk for violence perpetration in a cohort of survivors of violence. This association was detectable even though neighborhood deprivation levels were higher than the median for the full sample of survivors.

There are realistically a vast complexity of social phenomena that may underlie this finding, but theoretical explanations introduced in sociological and anthropological literature offer key insights. First, residing in a more resource-deprived and sociopolitically marginalized neighborhood may be associated with increased likelihood that someone injured by violence will use violence to maintain respect and social capital, with the intention of assuring physical safety for the individual and loved ones.^51,52^ This “code of the street,” as dubbed by ethnographer Elijah Anderson,^53^ may encourage retaliation against the person who perpetrated the injury or the demonstration of strength and capacity for violence by using it against a third party. The spatial concentration of these types of social forces follows from historical and contemporary forms of racialized economic marginalization, such as urban manufacturing job loss and the criminalization of Black youth^54^; thus, they would be expected to be associated with ICE-based deprivation measures. Our finding may also be explained by the overpolicing of neighborhoods with high rates of Black poverty (ie, higher deprivation levels).^54,55,56^ Individuals residing in neighborhoods with higher-deprivation levels may have been more likely to encounter police and be charged with violence perpetration, irrespective of these individuals’ use of violence.

Conversely, we found that the level of neighborhood deprivation was not associated with the risk of violent reinjury in our cohort of survivors of violence. This was a surprising result given that previous studies^15,51^ found that neighborhood factors, including high rates of poverty, violence, and illicit drug use and sales, were associated with violent injuries and reinjury. However, a history of violent injury was previously found to be the factor associated with the greatest increase in recurrent intentional injuries.^25,57^ It is possible that we were not able to discern a significant increase in violent reinjury by level of neighborhood deprivation because our cohort was preselected for individuals at highest risk of reinjury (ie, individuals who previously experienced violence from neighborhoods with deprivation levels higher than the median). Another explanation may be that residential location provides an incomplete measure of daily activity space (ie, the locations where people spend their time).^58^ Future work could examine this question by combining information about where individuals lived and where they were injured. By contrast, a substance use disorder prior to the initial violent injury was associated with reinjury, suggesting that treatment programs for substance use disorders may be a valuable part of reinjury prevention.

HVIPs are designed to seize upon the window of opportunity after a traumatic injury, when an individual who experienced violence is at a crossroads and may be more amenable to intervention.^13,14^ For those who have experienced violence, this intervention may alter their life trajectory toward a path away from violence and injury. Our results, with an understanding of the limitations of our police-reported data, suggest that HVIPs should consider the first year after injury as the most critical period for intensive interventions geared toward clients at highest risk for violence perpetration. After this first year, risk for violence perpetration decreases, while risk for reinjury remains more constant through the following 2 years, suggesting that this risk should remain a long-term priority for HVIPs.

However, there are limits to what HVIPs can achieve in reducing community violence within the constraints of limited client engagement^23^ and structural racism and neighborhood disinvestment^59^ in areas where most survivors of community violence reside. In future analyses, it may be important to assess the interaction of HVIP engagement and client services with the association between racialized economic segregation and violence perpetration as a successful means for breaking the cycle of community violence. Notably, HVIP services related to housing and relocation have proved to be some of the more challenging needs to fulfill for individuals in HVIPs,^19,23^ suggesting that helping survivors relocate to less-deprived areas is unlikely to be a scalable solution. Instead, it is likely that a range of social and structural determinants of health in a neighborhood must be addressed for survivors of violence to overcome neighborhood-level factors associated with postinjury violent outcomes. Combating the root causes of violence in segregated urban communities is critical to ensuring safety for individuals who have survived injuries, with particular consideration for societal conditions stemming from structural racism. This effort can be achieved only through partnerships among HVIPs, community organizations, and local and federal governments.

### Limitations

This study has several limitations. First, our population included patients treated at a single medical center, which limits the generalizability of our findings to other patient populations in other US cities. Second, while only patients with a known home address after hospital discharge were included in this study, we cannot be certain that patients actually resided at the listed addresses, and we had no way to continually follow up with patients if they moved residences, experienced homelessness, or became incarcerated during the study period. However, we have accounted for neighborhood-level residential instability in our models. Third, data regarding violence perpetration were acquired from BPD reports. There were likely additional acts of violence perpetration in this population that were not reported to police^60^ or not included in police reports. Further, the well-documented^61,62^ disparate police practices in urban White and Black neighborhoods suggest that communities with predominantly racial and ethnic minority populations and higher levels of poverty and violence may be policed at higher rates than more privileged neighborhoods. Thus, increased police surveillance in more deprived neighborhoods could partly account for the association we observed between racialized economic segregation and violence perpetration. Fourth, data regarding violent reinjury were acquired from BMC hospital records, Boston police reports, and Massachusetts death records. Recurrent violent injuries treated at other Boston hospitals or those unknown to Boston police or occurring outside the state or metro area were not captured in this analysis.

## Conclusions

In this retrospective cohort study, we observed that survivors of violence living in more deprived neighborhoods were at increased risk for postinjury violence perpetration. The level of neighborhood deprivation was not associated with the risk of violent reinjury in our population. Patients with the highest risk for alleged violence perpetration were younger and more frequently men and Black, while those with a baseline diagnosis of substance use were at highest risk for violent reinjury. Our results suggest that HVIPs should consider the first year after injury as the most crucial period for interventions directed toward clients at highest risk for violence perpetration. We found that after this first year, occurrence of violence perpetration decreased while the rate of reinjury remained more constant, suggesting that reinjury should continue to be a long-term priority for HVIPs. This study found that racialized poverty in urban communities was associated with adverse outcomes among survivors of violence, underscoring the importance of addressing the root causes of violence, with particular consideration for societal conditions stemming from structural racism.

## Notes

Supplement 1. **eFigure 1.** Flowchart of Analysis **eFigure 2.** Histogram of Patients Treated for a Violent Penetrating Injury at Boston Medical Center by Level of Neighborhood Deprivation, 2013-2018 **eTable 1.** Frequencies of Police-Reported Violence Perpetration and Violent Reinjury at 3 Years After a Violent Penetrating Injury Treated at Boston Medical Center, 2013-2021 **eTable 2.** Risk of Police-Reported Violence Perpetration Within 3 Years After Surviving a Penetrating Injury Treated at Boston Medical Center, 2013-2021 **eTable 3.** Risk of Violent Reinjury Within 3 Years After Surviving a Penetrating Injury Treated at Boston Medical Center, 2013-2021 **eTable 4.** Sensitivity Analysis of Risk of Violence at 1 and 3 Years After Surviving a Penetrating Injury by Level of Neighborhood Deprivation, 2013-2021 **eTable 5.** Baseline Neighborhood Characteristics for Individuals Experiencing Penetrating Injury Treated at Boston Medical Center, 2013-2018 **eFigure 3.** Cumulative Incidence of Violent Reinjury by Level of Neighborhood Deprivation, 2013-2021 **eAppendix 1.** Study Design **eAppendix 2.** Covariates **eAppendix 3.** Exposure **eAppendix 4.** Outcomes **eAppendix 5.** Data Analysis Methods
eReferences.

Supplement 2.
Data Sharing Statement
