# Supplementary material for: Exposure to Neighborhood Racialized Economic Segregation and Reinjury and Violence Perpetration Among Survivors of Violent Injuries
Source: JAMA Netw Open. 2023 Apr 26;6(4):e238404. doi: 10.1001/jamanetworkopen.2023.8404 (PMC10134006; doi:10.1001/jamanetworkopen.2023.8404)
Supplement: Supplement 1. — eFigure 1. Flowchart of Analysis eFigure 2. Histogram of Patients Treated for a Violent Penetrating Injury at Boston Medical Center by Level of Neighborhood Deprivation, 2013-2018 eTable 1. Frequencies of Police-Reported Violence Perpetration and Violent Reinjury at 3 Years After a Violent Penetrating Injury Treated at Boston Medical Center, 2013-2021 eTable 2. Risk of Police-Reported Violence Perpetration Within 3 Years After Surviving a Penetrating Injury Treated at Boston Medical Center, 2013-2021 eTable 3. Risk of Violent Reinjury Within 3 Years After Surviving a Penetrating Injury Treated at Boston Medical Center, 2013-2021 eTable 4. Sensitivity Analysis of Risk of Violence at 1 and 3 Years After Surviving a Penetrating Injury by Level of Neighborhood Deprivation, 2013-2021 eTable 5. Baseline Neighborhood Characteristics for Individuals Experiencing Penetrating Injury Treated at Boston Medical Center, 2013-2018 eFigure 3. Cumulative Incidence of Violent Reinjury by Level of Neighborhood Deprivation, 2013-2021 eAppendix 1. Study Design eAppendix 2. Covariates eAppendix 3. Exposure eAppendix 4. Outcomes eAppendix 5. Data Analysis Methods eReferences. [file jamanetwopen-e238404-s001.pdf]

# Supplemental Online Content

Pino EC, Jacoby SF, Dugan E, Jay J. Exposure to neighborhood racialized economic segregation and reinjury and violence perpetration among survivors of violent injuries. *JAMA Netw Open.* 2023;6(4):e238404. doi:10.1001/jamanetworkopen.2023.8404

**eFigure 1.** Flowchart of Analysis

**eFigure 2.** Histogram of Patients Treated for a Violent Penetrating Injury at Boston Medical Center by Level of Neighborhood Deprivation, 2013-2018

**eTable 1.** Frequencies of Police-Reported Violence Perpetration and Violent Reinjury at 3 Years After a Violent Penetrating Injury Treated at Boston Medical Center, 2013-2021

**eTable 2.** Risk of Police-Reported Violence Perpetration Within 3 Years After Surviving a Penetrating Injury Treated at Boston Medical Center, 2013-2021

**eTable 3.** Risk of Violent Reinjury Within 3 Years After Surviving a Penetrating Injury Treated at Boston Medical Center, 2013-2021

**eTable 4.** Sensitivity Analysis of Risk of Violence at 1 and 3 Years After Surviving a Penetrating Injury by Level of Neighborhood Deprivation, 2013-2021

**eTable 5.** Baseline Neighborhood Characteristics for Individuals Experiencing Penetrating Injury Treated at Boston Medical Center, 2013-2018

**eFigure 3.** Cumulative Incidence of Violent Reinjury by Level of Neighborhood Deprivation, 2013-2021

**eAppendix 1.** Study Design

**eAppendix 2.** Covariates

**eAppendix 3.** Exposure

**eAppendix 4.** Outcomes

**eAppendix 5.** Data Analysis Methods

**eReferences**

This supplemental material has been provided by the authors to give readers additional information about their work.

© 2023 Pino EC et al. *JAMA Network Open.*

**eFigure 1.** Flowchart of Analysis

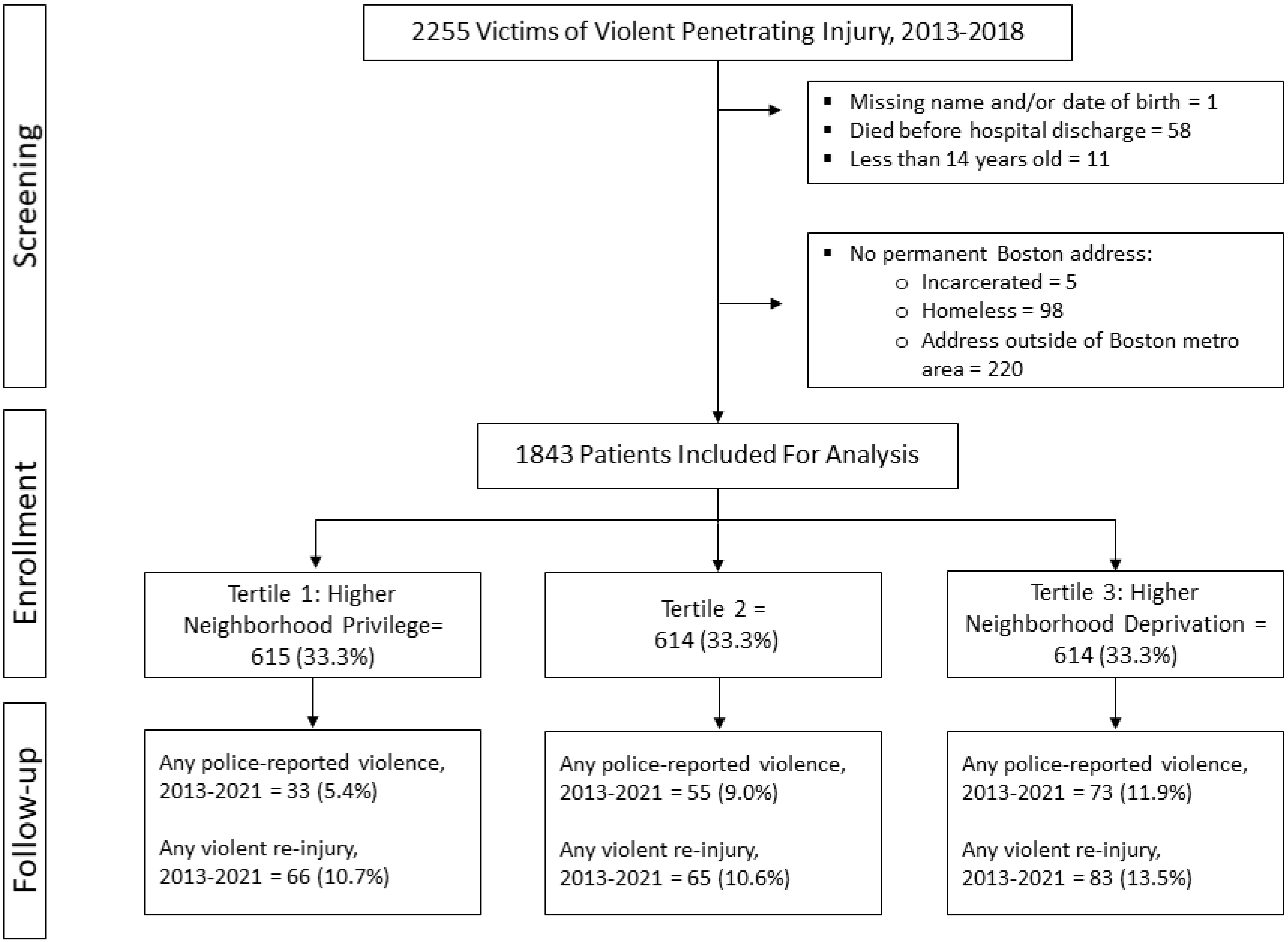


© 2023 Pino EC et al. *JAMA Network Open.*

**eFigure 2**. Histogram of Patients Treated for a Violent Penetrating Injury at Boston Medical Center by Level of Neighborhood Deprivation, 2013-2018

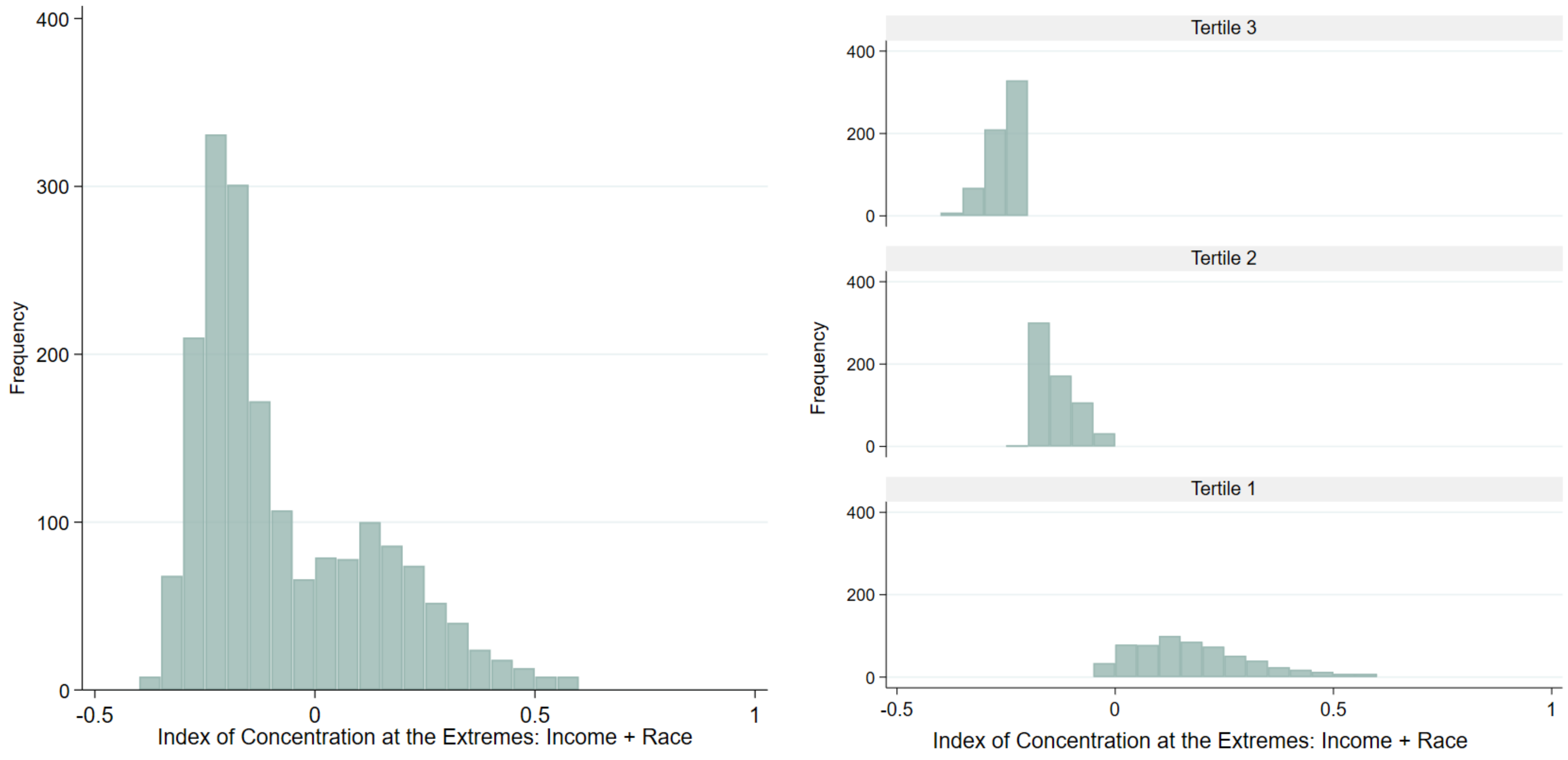


Inverse distance weighted Index of Concentration at the Extremes (ICE) measures for racial and economic segregation were calculated for each patient home address at hospital discharge. ICE scores range from −1 (most deprived) to 1 (most privileged).

© 2023 Pino EC et al. *JAMA Network Open.*

**eTable 1.** Frequencies of Police-Reported Violence Perpetration and Violent Reinjury at 3 Years After a Violent Penetrating Injury Treated at Boston Medical Center, 2013-2021

| | Overall, n (%) | Neighborhood Deprivation | | |
|---|---|---|---|---|
| | | Tertile 1, n (%) | Tertile 2, n (%) | Tertile 3, n (%) |
| **In year 1** | | | | |
| **Any violence perpetration** | **96 (5.2)** | **19 (3.1)** | **29 (4.7)** | **48 (7.8)** |
| Stab wound | 7 (0.4) | 1 (0.2) | 3 (0.5) | 3 (0.5) |
| Gunshot wound | 9 (0.5) | 2 (0.3) | 4 (0.7) | 3 (0.5) |
| Blunt assault | 51 (2.8) | 11 (1.8) | 15 (2.4) | 25 (4.1) |
| Threaten without assault | 29 (1.6) | 5 (0.8) | 7 (1.1) | 17 (2.8) |
| **Any violent re-injury** | **112 (6.1)** | **37 (6.0)** | **27 (4.4)** | **48 (7.8)** |
| Stab wound | 22 (1.2) | 5 (0.8) | 7 (1.1) | 10 (1.6) |
| Gunshot wound | 36 (2.0) | 9 (1.5) | 9 (1.5) | 18 (2.9) |
| Assault | 54 (2.9) | 23 (3.7) | 11 (1.8) | 20 (3.3) |
| **In year 2** | | | | |
| **Any violence perpetration** | **42 (2.4)** | **9 (1.5)** | **18 (3.1)** | **15 (2.7)** |
| Stab wound | 2 (0.1) | 0 (0.0) | 0 (0.0) | 2 (0.4) |
| Gunshot wound | 4 (0.2) | 0 (0.0) | 4 (0.7) | 0 (0.0) |
| Blunt assault | 22 (1.3) | 6 (1.0) | 9 (1.6) | 7 (1.3) |
| Threaten without assault | 14 (0.8) | 3 (0.5) | 5 (0.9) | 6 (1.1) |
| **Any violent re-injury** | **62 (3.6)** | **18 (3.1)** | **20 (3.4)** | **24 (4.3)** |
| Stab wound | 16 (0.9) | 5 (0.9) | 3 (0.5) | 8 (1.4) |
| Gunshot wound | 23 (1.3) | 4 (0.7) | 11 (1.9) | 8 (1.4) |
| Assault | 23 (1.3) | 9 (1.6) | 6 (1.0) | 8 (1.4) |
| **In year 3** | | | | |
| **Any violence perpetration** | **23 (1.1)** | **5 (0.9)** | **8 (1.4)** | **10 (1.8)** |
| Stab wound | 4 (0.2) | 2 (0.3) | 1 (0.2) | 1 (0.2) |
| Gunshot wound | 4 (0.2) | 1 (0.2) | 2 (0.4) | 1 (0.2) |
| Blunt assault | 11 (0.7) | 1 (0.2) | 4 (0.7) | 6 (1.1) |
| Threaten without assault | 4 (0.2) | 1 (0.2) | 1 (0.2) | 2 (0.4) |
| **Any violent re-injury** | **42 (2.5)** | **11 (2.0)** | **15 (2.7)** | **16 (3.0)** |
| Stab wound | 14 (0.8) | 3 (0.5) | 4 (0.7) | 7 (1.3) |
| Gunshot wound | 12 (0.7) | 3 (0.5) | 4 (0.7) | 5 (0.9) |
| Assault | 16 (1.0) | 5 (0.9) | 7 (1.3) | 4 (0.7) |
| **Cumulative at 3 years** | | | | |
| **Any violence perpetration** | **161 (8.7)** | **33 (5.4)** | **55 (9.0)** | **73 (11.9)** |
| Stab wound | 14 (0.8) | 3 (0.5) | 5 (0.8) | 6 (1.0) |
| Gunshot wound | 17 (0.9) | 3 (0.5) | 10 (1.6) | 4 (0.7) |
| Blunt assault | 84 (4.6) | 18 (2.9) | 28 (4.6) | 38 (6.2) |
| Threaten without assault | 46 (2.5) | 9 (1.5) | 12 (2.0) | 25 (4.1) |
| **Any violent re-injury** | **214 (11.6)** | **66 (10.7)** | **65 (10.6)** | **83 (13.5)** |
| Stab wound | 50 (2.7) | 13 (2.1) | 17 (2.8) | 20 (3.3) |
| Gunshot wound | 71 (3.9) | 16 (2.6) | 24 (3.9) | 31 (5.1) |
| Assault | 93 (5.1) | 37 (6.0) | 24 (3.9) | 32 (5.2) |

All values are frequencies and column percentages. Threats of violence include verbal threats, threatening texts or phone messages, or attempted assault, stabbing, or shooting.

© 2023 Pino EC et al. *JAMA Network Open.*

**eTable 2.** Risk of Police-Reported Violence Perpetration Within 3 Years After Surviving a Penetrating Injury Treated at Boston Medical Center, 2013-2021

| **Any Perpetration of Violence** | | **Crude Model** | | **Full Multivariable Model** | | **Final Multivariable Model** | |
|---|---|---|---|---|---|---|---|
| | **n (%)** | **HR (95% CI)** | ***p*** | **HR (95% CI)** | ***p*** | **HR (95% CI)** | ***p*** |
| **Total incidents** | **161 (8.7)** | | | | | | |
| ICE Deprivation Index | | 1.23 (1.12-1.35) | <0.0001 | 1.13 (1.02-1.24) | 0.02 | 1.13 (1.03-1.25) | 0.01 |
| Injury Year | | 0.72 (0.65-0.80) | <0.0001 | 0.77 (0.69-0.85) | <0.0001 | 0.76 (0.68-0.84) | <0.0001 |
| Age | | 0.92 (0.90-0.94) | <0.0001 | 0.93 (0.91-0.96) | <0.0001 | 0.93 (0.91-0.95) | <0.0001 |
| Gender | | | 0.0001 | | 0.0003 | | 0.0001 |
| Female | 5 (1.8) | 1.00 | | 1.00 | | 1.00 | |
| Male | 156 (10.0) | 6.04 (2.48-14.71) | | 5.28 (2.15-12.99) | | 5.95 (2.44-14.49) | |
| Race/ethnicity | | | 0.0002, 0.0001 | | 0.0002, 0.0002 | | 0.0008, 0.0003 |
| Black | 145 (11.4) | 17.71 (2.48-126.61) | | 9.52 (1.31-68.99) | | 10.60 (1.47-76.59) | |
| Hispanic | 15 (4.3) | 6.37 (0.84-48.26) | | 3.52 (0.46-26.81) | | 3.79 (0.21-28.83) | |
| White | 1 (0.7) | 1.00 | | 1.00 | | 1.00 | |
| Other | 0 (0.0) | - | | - | | - | |
| Unknown | 0 (0.0) | - | | - | | - | |
| Insurance Payer | | | 0.77, 0.78 | | 0.80, 0.61 | | |
| Medicaid/ Medicare | 94 (8.7) | 1.00 | | 1.00 | | | |
| Private | 15 (9.4) | 1.07 (0.62-1.85) | | 1.19 (0.68-2.06) | | | |
| No health insurance | 20 (10.2) | 1.19 (0.73-1.92) | | 1.24 (0.76-2.02) | | | |
| Unknown | 32 (7.8) | 0.88 (0.59-1.32) | | 1.10 (0.73-1.65) | | | |
| Employment Status | | | 0.01, 0.004 | | 0.17, 0.08 | | |
| Employed | 43 (6.7) | 1.00 | | 1.00 | | | |
| Unemployed | 80 (11.2) | 1.72 (1.19-2.50) | | 1.40 (0.96-2.04) | | | |
| Unknown | 38 (7.8) | 1.17 (0.76-1.81) | | 1.43 (0.91-2.25) | | | |
| **Co-morbidities** | | | | | | | |
| Any substance use /abuse | 14 (3.8) | 0.38 (0.22-0.65) | 0.0005 | 0.82 (0.46-1.46) | 0.50 | | |
| Any mental health disorder | 13 (6.0) | 0.65 (0.37-1.15) | 0.14 | 0.89 (0.50-1.58) | 0.69 | | |
| **Injury Specifics** | | | | | | | |
| Injury Type | | | <0.0001 | | 0.09 | | |
| Stab Wound | 58 (5.5) | 1.00 | | 1.00 | | | |
| Gunshot Wound | 103 (13.0) | 2.43 (1.76-3.36) | | 1.35 (0.96-1.90) | | | |
| **Sensitivity Analyses** | | | | | | | |
| Residential Instability | | 0.99 (0.98-1.00) | 0.17 | 1.00 (0.98-1.01) | 0.84 | | |
| Distance to Level I Trauma Center | | 0.93 (0.86-1.00) | 0.06 | 0.97 (0.89-1.06) | 0.54 | | |

© 2023 Pino EC et al. *JAMA Network Open.*

Cox proportional hazards regression models were used to estimate hazard ratios (HR) and 95% confidence intervals (CI). Full multivariable model is adjusted for Index of Concentration at the Extremes (ICE) deprivation score, age, gender, race/ethnicity, insurance payer, employment status, history of substance use/abuse, history of mental health diagnosis, injury type, and injury year. Final multivariable model is adjusted for ICE deprivation score, age, gender, race/ethnicity, and year of initial injury. ICE Deprivation Index HRs are reported as the change in risk per 0.1 unit change in ICE neighborhood racial segregation and economic deprivation on a scale from 1 to -1. "Other" race includes Asian and all other races.

* *p*-value excluding unknown category.

© 2023 Pino EC et al. *JAMA Network Open.*

**eTable 3.** Risk of Violent Reinjury Within 3 Years After Surviving a Penetrating Injury Treated at Boston Medical Center, 2013-2021

| **Any Violent Re-Injury** | | **Crude Model** | | **Full Multivariable Model** | | **Final Multivariable Model** | |
|---|---|---|---|---|---|---|---|
| | **n (%)** | **HR (95% CI)** | ***p*** | **HR (95% CI)** | ***p*** | **HR (95% CI)** | ***p*** |
| **Total re-injuries** | **214 (11.6)** | | | | | | |
| ICE Deprivation Index | | 1.04 (0.97-1.11) | 0.30 | 1.03 (0.96-1.11) | 0.39 | 1.03 (0.96-1.11) | 0.38 |
| Injury year | | 0.93 (0.86-1.01) | 0.09 | 0.96 (0.89-1.05) | 0.40 | 0.95 (0.87-1.03) | 0.18 |
| Age | | 1.00 (0.99-1.01) | 0.62 | 0.99 (0.98-1.00) | 0.20 | 0.99 (0.98-1.00) | 0.16 |
| Gender | | | 0.40 | | 0.22 | | 0.32 |
| Female | 29 (10.1) | 1.00 | | 1.00 | | 1.00 | |
| Male | 185 (11.9) | 1.18 (0.80-1.75) | | 1.28 (0.86-1.92) | | 1.22 (0.83-1.81) | |
| Race/ethnicity | | | 0.07, 0.04* | | 0.11, 0.11* | | 0.17, 0.09* |
| Black | 164 (12.9) | 1.00 | | 1.00 | | 1.00 | |
| Hispanic | 28 (8.0) | 0.61 (0.41-0.91) | | 0.65 (0.43-0.98) | | 0.64 (0.42-0.96) | |
| White | 21 (14.1) | 1.12 (0.71-1.77) | | 1.04 (0.63-1.71) | | 1.03 (0.63-1.70) | |
| Other | 1 (3.0) | 0.23 (0.03-1.62) | | 0.27 (0.04-1.91) | | 0.28 (0.04-1.99) | |
| Unknown | 0 (0.0) | - | | - | | - | |
| Insurance payer | | | 0.10, 0.12* | | 0.24, 0.44* | | |
| Medicaid/ Medicare | 141 (13.1) | 1.00 | | 1.00 | | | |
| Private | 12 (7.6) | 0.56 (0.31-1.00) | | 0.68 (0.37-1.23) | | | |
| No health insurance | 21 (10.7) | 0.81 (0.51-1.28) | | 0.92 (0.58-1.47) | | | |
| Unknown | 40 (9.7) | 0.73 (0.51-1.04) | | 0.72 (0.50-1.04) | | | |
| Employment Status | | | 0.001, 0.004* | | 0.005, 0.08 | | |
| Employed | 51 (8.0) | 1.00 | | 1.00 | | | |
| Unemployed | 91 (12.8) | 1.65 (1.17-2.32) | | 1.37 (0.96-1.94) | | | |
| Unknown | 72 (14.7) | 1.93 (1.35-2.76) | | 1.89 (1.29-2.77) | | | |
| **Co-morbidities** | | | | | | | |
| Any substance use /abuse | 73 (20.0) | 2.25 (1.70-2.98) | <0.0001 | 2.27 (1.64-3.13) | <0.0001 | 2.38 (1.74-3.26) | <0.0001 |
| Any mental health disorder | 30 (13.9) | 1.24 (0.84-1.83) | 0.27 | 1.16 (0.78-1.72) | 0.45 | | |
| **Injury Specifics** | | | | | | | |
| Injury Type | | | 0.92 | | 0.28 | | |
| Stab Wound | 122 (11.6) | 1.00 | | 1.00 | | | |
| Gunshot Wound | 92 (11.6) | 0.99 (0.75-1.29) | | 1.18 (0.87-1.60) | | | |
| **Sensitivity Analyses** | | | | | | | |
| Residential Instability | | 1.00 (0.99-1.01) | 0.99 | 1.00 (0.99-1.02) | 0.74 | | |
| Distance to Level I Trauma Center | | 0.94 (0.88-1.00) | 0.07 | 0.95 (0.89-1.02) | 0.15 | | |

© 2023 Pino EC et al. *JAMA Network Open.*

Cox proportional hazards regression models were used to estimate hazard ratios (HR) and 95% confidence intervals (CI). Full multivariable model is adjusted for Index of Concentration at the Extremes (ICE) deprivation score, age, gender, race/ethnicity, insurance payer, history of substance use/abuse, history of mental health diagnosis, injury type, and injury year. Final multivariable model is adjusted for ICE deprivation score, age, race/ethnicity, gender, history of substance use/abuse, and injury year. ICE Deprivation Index HRs are reported as the change in risk per 0.1-unit increase in neighborhood deprivation. * *p*-value excluding unknown category. "Other" race includes Asian and all other races.

© 2023 Pino EC et al. *JAMA Network Open.*

**eTable 4.** Sensitivity Analysis of Risk of Violence at 1 and 3 Years After Surviving a Penetrating Injury by Level of Neighborhood Deprivation, 2013-2021

| **Sensitivity Analysis** | **Police-reported violence perpetration** | | | **Violent re-injury** | | |
|---|---|---|---|---|---|---|
| **n = 1640 (203 excluded for addresses outside Boston)** | **n (%)** | **Multivariable Model HR (95%CI)** | ***p*** | **n (%)** | **Multivariable Model HR (95%CI)** | ***p*** |
| **At 1 year** | **84 (5.1)** | | | **103 (6.3)** | | |
| ICE Deprivation Index | | 1.23 (1.04-1.46) | 0.01 | | 1.05 (0.94-1.18) | 0.37 |
| **At 3 years** | **146 (8.9)** | | | **194 (11.8)** | | |
| ICE Deprivation Index | | 1.14 (1.02-1.28) | 0.03 | | 1.06 (0.98-1.16) | 0.16 |

© 2023 Pino EC et al. *JAMA Network Open.*

**eTable 5.** Baseline Neighborhood Characteristics for Individuals Experiencing Penetrating Injury Treated at Boston Medical Center, 2013-2018

| Neighborhood Characteristics | Index of Concentration at the Extremes (ICE) | | | | *P* |
|---|---|---|---|---|---|
| | Overall | Tertile 1: Higher Privilege | Tertile 2 | Tertile 3: Higher Deprivation | |
| **No. of patients (row percentage)** | **1843** | **615 (33.3)** | **614 (33.3)** | **614 (33.3)** | |
| Residential instability[a], median (IQR) | 40 (33-47) | 44 (33-53) | 41 (33-49) | 37 (29-41) | 0.0001 |
| Distance to level I trauma center, median kilometers (IQR) | 2.0 (1.4-2.8) | 3.0 (1.5-6.6) | 2.0 (1.3-2.5) | 1.8 (1.3-2.4) | 0.0001 |

[a] Residential instability refers to the percentage of households in a census tract that moved between 2015 and 2021 according to the 2021 5-year American Community Survey (ACS) data.

© 2023 Pino EC et al. *JAMA Network Open.*

**eFigure 3.** Cumulative Incidence of Violent Reinjury by Level of Neighborhood Deprivation, 2013-2021

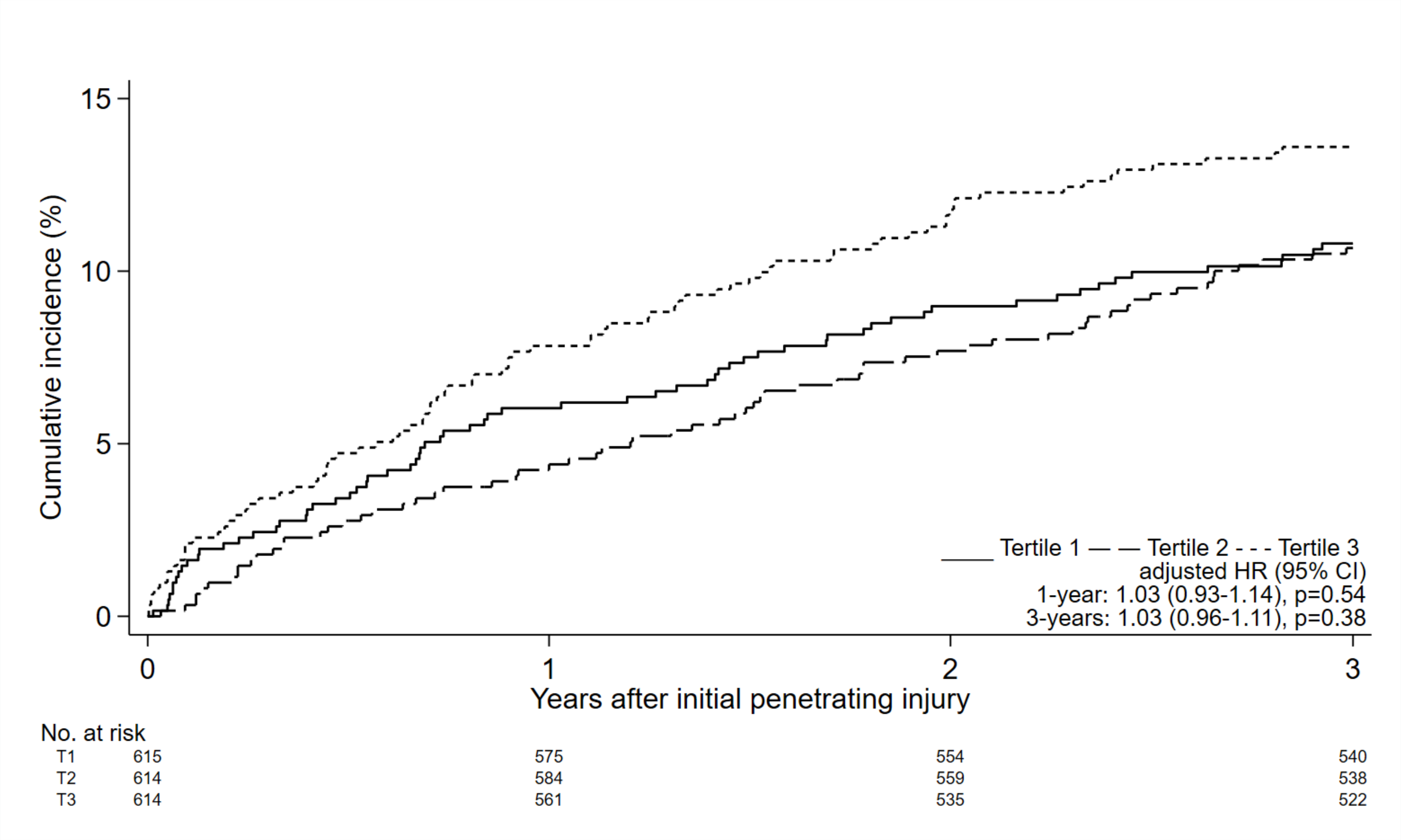


The Index of Concentration at the Extremes (ICE) is assessed as a continuous variable. All hazard ratios (HRs) and 95% confidence intervals (95% CI) are reported as the change in risk per 0.1-unit change in ICE racialized economic segregation on a scale from 1 to -1.

© 2023 Pino EC et al. *JAMA Network Open.*

**eAppendix 1.** Study Design

Injuries deemed to be self-inflicted were excluded. Although we refer to the baseline injury for this analysis as an "index" injury, it is likely that many patients' history with violence—as victim, perpetrator, or witness—spans years before this incident.[1] Data included in this analysis originates from electronic health records, trauma registries, the BMC Clinical Data Warehouse, the 2013 to 2021 Massachusetts Death File from the Registry of Vital Records and Statistics, and Boston Police Department (BPD) reports from the Boston Regional Intelligence Center (BRIC). Boston police reports from BRIC include names and dates of birth for involved individuals, and incident details for property and violent crimes in Boston, including fatal and non-fatal penetrating injuries and assaults, firearm arrests, robberies, burglaries, vehicle crimes and drug incidents.

Criteria for inclusion in this analysis required complete data for name and date of birth and survival to hospital discharge, which allowed for determination of follow-up outcomes. Patients less than 14 years of age were excluded as no police-reported incidents of violence perpetration were recorded children of this age group. Patients with no home address (i.e. those experiencing homelessness or incarceration) upon discharge were excluded. Patients with a residential address farther than 15 miles from the geographic center of Boston were excluded, as there was a decreased likelihood that incidents of violence perpetration would be captured by BPD for patients outside this radius.

© 2023 Pino EC et al. *JAMA Network Open.*

**eAppendix 2.** Covariates

Age was assessed as a continuous variable and dichotomized into categories by the median patient age of 26 years. All patients were grouped with their affirmed gender for all analyses, including six transgender females and one transgender male. We abstracted the self-identified race and ethnicity from the patient medical record. Race/ethnicity was classified into five categories: non-Hispanic White, non-Hispanic Black, Hispanic (any race), other race/ethnicity which includes all other races, and those missing race/ethnicity information ("unknown"). Health insurance payer, which served as a proxy for individual-level socioeconomic status, was classified into four categories: Medicaid/Medicare, private, no health insurance, and unknown insurance status. Employment status was classified by self-report as either employed (including both reported and unreported employment) or unemployed (including those seeking or not seeking work, students, retirees, and those unable to work due to disability or immigration status). Additional covariates were substance use disorders (alcohol, cocaine, heroin/opioids, or other type of illicit drug use disorder), and mental health disorders (depression, anxiety, suicidal/homicidal ideation, PTSD, bipolar disorder, schizoaffective disorder/schizophrenia/psychosis, mood/personality disorder), coded as binary. Data on demographics and comorbidities were obtained from electronic health records and self-report.

Index penetrating injuries were categorized by injury type (gunshot wound, stab wound), bodily location of injury, and hospital disposition. Of those patients who were admitted to the hospital, we assessed the length of stay, discharge placement, disability status, and injury severity scores (ISS).

Residential instability refers to the percentage of households in a census tract that moved residences between 2015 and 2021 according to the 2021 5-year American Community Survey (ACS) data (ACS table B25038). This variable is included as a proxy measure for the likelihood that a patient could move residences during the study period. The distance from patients' residence to the closest level I trauma center is assessed in kilometers and included in analyses to test for potential differences in capturing re-injury data for patients residing farther from high-level trauma care.

**eAppendix 3.** Exposure

Patients' deprivation exposure was assigned based on the residential address they provided at intake in the emergency department or while inpatient in the hospital and corroborated when possible with VIAP records. The race-income ICE measure relied on census tract data obtained from the 2013-2017 American Community Survey (ACS). ICE was calculated by subtracting the number of Black-headed households with incomes below the poverty line from the number of White-headed, affluent households (income greater than $100,000 year), then dividing this quantity by the total number of households.[2]

Census tracts are the smallest spatial units for which ICE can be calculated from intercensal (*i.e.,* ACS) data. However, census tracts are relatively arbitrary spatial units, so it was not appropriate to assign a single ICE level to every address in the same census tract. Instead, address-level ICE scores were assigned using inverse distance weighting from census tract centroids, with a power of 2 and a half-mile bandwidth. This spatial interpolation approach has been used in prior studies of urban violence.[3] For a small proportion of addresses (3%, N = 61), predominantly located in less densely settled areas surrounding the City of Boston, no census tract centroid fell within ½ mile. For these, we used the same algorithm with no maximum bandwidth parameter.

© 2023 Pino EC et al. *JAMA Network Open.*

**eAppendix 4.** Outcomes

Violent re-injuries included any stab wounds, gunshot wounds, or blunt assaults treated at Boston Medical Center and/or involved Boston police interaction. Police-reported violence perpetration included any stabbing, firearm injury, blunt assault, or threats of violence that involved the BPD. Threats of violence included verbal threats, threatening texts or phone messages, or attempted assault, stabbing, or shooting.

© 2023 Pino EC et al. *JAMA Network Open.*

**eAppendix 5.** Data Analysis Methods

One potential concern was residual spatial autocorrelation, which would indicate unmeasured confounding that biased parameter estimates. After fitting each regression as a generalized linear model, we used Global Moran's I tests to assess each model for residual spatial autocorrelation. Spatial neighborhoods were defined based on distance. The test was conducted using a range of possible distances, from 100 feet to 1 mile. When residual spatial autocorrelation was detected, we repeated the same regression as a generalized additive model, incorporating a thin plate regression spline over the X and Y coordinates.[4] This term is intended to capture the residual spatial trend, removing bias from the parameter estimates. This non-parametric approach has been used in prior injury research to improve the validity of regression models using spatially indexed data.[5,6]

Crude univariate estimates were derived for each covariate. All covariates were included in the full model. The final multivariable Cox regression model for police-reported violence perpetration was adjusted for significant covariates from the full model: age, gender, race/ethnicity, and year of initial injury (c-statistic = 0.80). The final multivariable Cox regression model for violent re-injury was adjusted for significant covariates from the full model: age, gender, race/ethnicity, year of initial injury, and history of substance use disorder (c-statistic = 0.65). Kaplan Meier curves for police-reported violence perpetration and violent re-injury were constructed after truncation at 3 years follow-up. For sensitivity analyses, we repeated these multivariable regression models only including patients with a Boston residential address, and assessed models after including variables for residential instability and distance to a level 1 trauma center. All analyses were conducted using R and Stata 16, and all maps were constructed in ArcGIS Pro.[7] Statistical tests used 2-sided $p<0.05$ as the threshold for significance.

© 2023 Pino EC et al. *JAMA Network Open.*

**eReferences**


1. Corbin TJ, Rich JA, Bloom SL, Delgado D, Rich LJ, Wilson AS. Developing a Trauma-Informed, Emergency Department–Based Intervention for Victims of Urban Violence. *J Trauma Dissociation.* 2011;12(5):510-525.
2. Feldman JM, Waterman PD, Coull BA, Krieger N. Spatial social polarisation: using the Index of Concentration at the Extremes jointly for income and race/ethnicity to analyse risk of hypertension. *Journal of epidemiology and community health.* 2015;69(12):1199-1207.
3. Branas CC, Kondo MC, Murphy SM, South EC, Polsky D, MacDonald JM. Urban Blight Remediation as a Cost-Beneficial Solution to Firearm Violence. *Am J Public Health.* 2016;106(12):2158-2164.
4. Wood SN. Thin plate regression splines. *J Roy Stat Soc Ser B (Stat Method).* 2003;65(1):95-114.
5. Goldstick JE, Lipton RI, Carter P, et al. The effect of neighborhood context on the relationship between substance misuse and weapons aggression in urban adolescents seeking ED care. *Subst Use Misuse.* 2015;50(5):674-684.
6. Jay J, Kondo MC, Lyons VH, Gause E, South EC. Neighborhood segregation, tree cover and firearm violence in 6 U.S. cities, 2015–2020. *Prev Med.* 2022:107256.
7. StataCorp. *Stata Statistical Software: Release 16* College Station, TX: StataCorp LLC; 2019.


© 2023 Pino EC et al. *JAMA Network Open.*
