# Supplementary material for: Exposure to Neighborhood Racialized Economic Segregation and Reinjury and Violence Perpetration Among Survivors of Violent Injuries
Source: JAMA Netw Open. 2023 Apr 26;6(4):e238404. doi: 10.1001/jamanetworkopen.2023.8404 (PMC10134006; doi:10.1001/jamanetworkopen.2023.8404)
Supplement: Supplement 2. — Data Sharing Statement [file jamanetwopen-e238404-s002.pdf]

Data Sharing Statement

Pino. Exposure to Neighborhood Racialized Economic Segregation and Reinjury and Violence Perpetration Among Survivors of Violent Injuries. *JAMA Netw Open*. Published online April 26, 2023. doi:10.1001/jamanetworkopen.2023.8404

Data
Data available: No

Additional Information
Explanation for why data not available: .

© 2023 Pino EC et al. *JAMA Network Open*.
